# Delivery Approaches for Therapeutic Genome Editing and Challenges

**DOI:** 10.3390/genes11101113

**Published:** 2020-09-23

**Authors:** Ilayda Ates, Tanner Rathbone, Callie Stuart, P. Hudson Bridges, Renee N. Cottle

**Affiliations:** 1 Department of Bioengineering, Clemson University, Clemson, SC 29634, USA; iates@g.clemson.edu (I.A.); trrathb@g.clemson.edu (T.R.); cdstuar@g.clemson.edu (C.S.); 2 College of Medicine, Medical University of South Carolina, Charleston, SC 29425, USA; bridges.hudson@gmail.com

**Keywords:** gene editing, delivery methods, CRISPR-Cas, ZFNs, TALENs, base editors, viral delivery, nonviral delivery

## Abstract

Impressive therapeutic advances have been possible through the advent of zinc-finger nucleases and transcription activator-like effector nucleases. However, discovery of the more efficient and highly tailorable clustered regularly interspaced short palindromic repeats (CRISPR) and associated proteins (Cas9) has provided unprecedented gene-editing capabilities for treatment of various inherited and acquired diseases. Despite recent clinical trials, a major barrier for therapeutic gene editing is the absence of safe and effective methods for local and systemic delivery of gene-editing reagents. In this review, we elaborate on the challenges and provide practical considerations for improving gene editing. Specifically, we highlight issues associated with delivery of gene-editing tools into clinically relevant cells.

## 1. Introduction

Gene-editing technologies have unprecedented potential to cure, treat, or prevent a wide array of inherited and acquired diseases. These technologies provide flexibility to modify the genome at precise locations for knockdown or restoration of gene expression, insertion of a therapeutic transgene, or correction of mutations associated with genetic diseases. Gene-editing-based clinical trials have advanced for multiple diseases.

Initiated in 2009, the first-in-human use of gene editing involved application of zinc-finger nucleases (ZFNs) to disrupt the HIV-1 major coreceptor, CCR5, in human CD4 T cells isolated from patients with chronic aviremic HIV infection (NCT00842634). To make the autologous CD4 cells resistant to HIV infection, 11% to 28% of alleles are modified with ZFNs. When infused back into the patients, the cells engrafted and showed long-term persistence. In one patient from the trial, HIV RNA was undetectable [1]. The results from this seminal study supported the feasibility and safety of ZFN-mediated genome editing in humans, laying the foundation for other gene-editing clinical trials. In a separate clinical trial for HIV (NCT02500849), the strategy involving CCR5-targeting ZFNs was used to introduce HIV-1-resistant mutations in autologous human CD34^+^ hematopoietic stem/progenitor cells (HSPCs) [2]. The first-in-human use of transcriptional activator-like effector nucleases (TALENs) entailed gene editing to make universal CAR T cells, UCART19, to treat children (NCT02808442) and adults (NCT02746952) with B cell acute lymphoblastic leukemia. In a small clinical trial in pediatric patients, the UCART19 cells were engineered to evade the lymphodepletion effects of anti-CD52 serotherapy, target B cell leukemia, and prevent graft-versus-host disease persistence in patients. The result was remissions after 28 days with subsequent successful allogeneic stem cell transplantation [3]. The first clinical trials involving in vivo gene editing have recently advanced to liver-directed therapies, namely mucopolysaccharidoses type 1 (NCT02702115) and type 2 (NCT03041324) and hemophilia B (NCT02695160).

Clustered regularly interspaced short palindromic repeats (CRISPR) and the associated protein (Cas9) nucleases represent the platform of choice for therapeutic applications. Cas9 nucleases have a simpler design, a wider targeting range, and provide higher levels of nuclease activity compared to the early generation of protein-based gene-editing tools. The first CRISPR clinical trial in the US was initiated in 2018 and involved use of autologous CD34^+^ HSPCs modified with CRISPR-Cas9 to upregulate the expression of fetal hemoglobin as a treatment for sickle cell disease and β-thalassemia (NCT03655678). In addition to hemoglobinopathies, clinical trials using CRISPR-mediated gene editing are underway for multiple cancers, such as gastrointestinal epithelial cancer (NCT04426669) and B cell malignancies (NCT04035434). Earlier this year, the first-in-human use of CRISPR gene editing in vivo occurred in clinical trials for the treatment of Leber congenital amaurosis-10, a hereditary retinal disease (NCT03872479). The CRISPR-based clinical trials are in early phases, and their results have not been reported in the literature.

Despite the recent clinical trials, a major barrier for therapeutic gene editing is the absence of safe and effective methods for local and systemic delivery of gene-editing reagents. In many diseases, such as Duchene muscular dystrophy, diseased tissue is widely distributed, necessitating in vivo delivery of gene-editing reagents. Further, because many diseases affect multiple body systems, a curative therapy must enable systemic delivery of the gene-editing reagents into multiple target cell types. This review aims to provide an overview of gene-editing tools and viral and nonviral delivery systems for therapeutic gene-editing applications. We elaborate on the strategies associated with each approach, the importance of delivery for effective therapies, challenges, and advances in the field. The discussed delivery systems with their advantages and disadvantages are summarized in Table 1.

## 2. Fundamentals of Gene Editing

**Figure 1:**
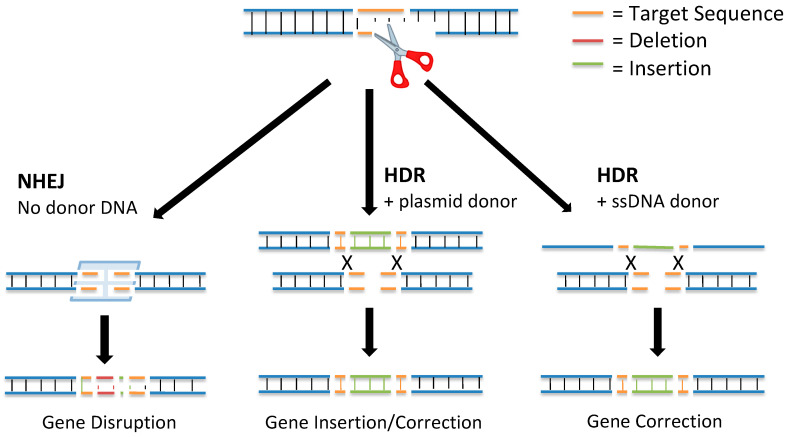
DNA repair pathways. Once a nuclease has been directed to the target sequence and has bound to the DNA strands, it induces a double-strand break (DSB). The DSB is repaired by endogenous cellular repair machinery through either the nonhomologous end joining (NHEJ) or homology-directed repair (HDR) pathway. NHEJ-mediated repair (shown on the left) results in random indels at the break site leading to gene disruption. Alternatively, HDR-mediated repair uses a donor template DNA, either a long double-stranded plasmid (center) or an ssDNA (right), and results in the incorporation of a desired genetic sequence for the correction or insertion of a gene. This figure has been adapted from [6].

DNA double-strand breaks (DSBs) activated at precise locations in the genome are the basis for gene editing using site-specific nucleases. DSBs are the most biologically damaging lesions; the absence of their repair can result in apoptosis, loss of genetic material, cell death, and permanent cell cycle arrest [4]. In some situations, DSBs can be programmed by the cell to correct errors introduced into the DNA during meiotic division or DNA replication. There are two main pathways used to repair DSBs (Figure 1): nonhomologous end joining (NHEJ) and homology-directed repair (HDR) [5].

In 1994, it was discovered that DSB repair mechanisms could be leveraged for genome editing [7]. By creating a DSB at a gene of interest and delivering a donor repair template, the gene could be targeted for editing through insertion of new DNA sequences via the HDR pathway. This discovery further revealed that new mutations could be introduced at the targeted gene through activation of a DSB and repair using the NHEJ pathway.

The NHEJ pathway is the most active DNA repair mechanism in mammalian cells. It repairs DSBs without a repair template [8]. The NHEJ DNA repair pathway is initiated when two complexes consisting of Mre11, Rad50, and Nbs1 bind to the severed ends. Once the DSB is detected, the two broken ends are held close together by DNA-dependent protein kinase subunits to allow DNA ligase IV to process the DNA and chemically seal the broken strands together, thus repairing the DSB [9]. The NHEJ mechanism of DNA repair is typically ≥ 70% accurate; however, through the processing of the damaged DNA using endonuclease activity, the NHEJ pathway can introduce a small insertion or deletion (indel) at the break site [10]. The presence of indels can disrupt gene function by causing a frameshift or exon skipping mutation [11]. In addition, the NHEJ repair pathway can be used to insert exogenous DNA sequences into the break site for targeted gene editing.

HDR is the more accurate DSB repair mechanism: It produces high fidelity repairs using a donor template DNA [8]. The HDR DNA repair mechanism begins with end-processing from the 5′ to 3′ ends by helicases and endonucleases. When a complementary repair template is found (often a sister chromatid, homologous chromosome, or other regions from the same chromosome), a strand from the DSB invades the template DNA and undergoes strand extension by DNA polymerase [5]. Then, the newly extended strand disassociates from the template and serves as a complement for the other strand on the DSB through a process called synthesis-dependent strand annealing. DNA synthesis occurs on the broken strand until the newly repaired DNA segments can be ligated to the original DNA strands at the site of the DSB [8].

During the HDR pathway, a donor DNA template can be used for targeted gene editing. Using the donor template, a sequence from the donor DNA is incorporated into the genome, resulting in edits ranging from single-nucleotide changes to insertions of multiple genes [12]. This method of precise gene editing has therapeutic potential in regenerative tissue or in a small number of edited cells that have the potential to improve disease symptoms [13].

In early studies, I-SceI, a homing nuclease isolated from *Saccharomyces cerevisiae* [14], and a homologous donor plasmid DNA were co-transfected into human COS-1 [7] and mouse embryonic cells [15]. The I-SceI made a DSB that would initiate the HDR repair pathway using the donor plasmid DNA as a repair template. This method of producing a knockin of a donor sequence was then tested in additional studies, confirming that the activation of a DSB using endonucleases along with the delivery of a donor DNA template results in the increased frequency of HDR by 3–5 orders of magnitude [16].While the use of I-SceI in studies helped to lay the foundation for gene editing, the low targeting specificity of the endonuclease limits its effectiveness as a gene-editing tool [17].

## 3. Gene-Editing Tools

**Figure 2:**
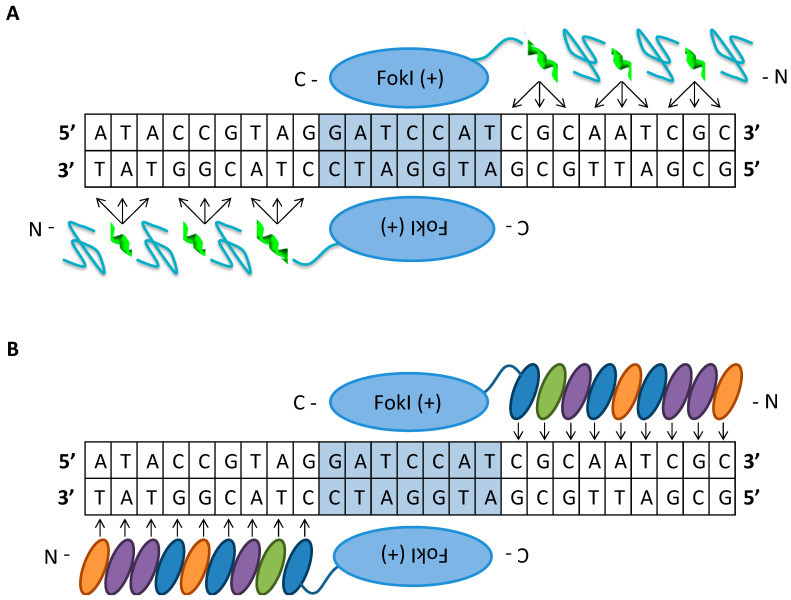
Protein-guided gene-editing tools. (**A**) A pair of zinc-finger nucleases comprised of an array of zinc-finger protein domains that bind to 3 nucleotides each, fused to a *FokI* endonuclease domain. The dimerization of two of these complexes on adjacent DNA strands in an inverted orientation generates a DSB at a site determined by the protein DNA binding domains. (**B**) A pair of transcriptional activator-like effector nucleases (TALEN) comprised of repeating TALE domains that each bind to a single nucleotide. These complexes are fused to a *FokI* endonuclease domain that binds to opposing DNA strands and arranged in an inverted tail-to-tail orientation, with optimized spacing determined by the TALE binding domains. Dimerization of these complexes enables the *FokI* domains to activate a DSB at the target site. This figure has been adapted from [6].

Site-specific nucleases have unprecedented potential to treat a wide array of inherited and acquired diseases. The objective for gene-editing-based gene therapy is the transformation of a native DNA sequence with a desired modified sequence within the genome of target cells for correction of a mutation, disruption of gene expression, deletion to restore the reading frame, or insertion of a new gene sequence to treat a disease. To elicit a therapeutic effect, heterozygous gene modification may be sufficient to ameliorate symptoms and quality of life in patients suffering from genetic or acquired diseases. The first generation of engineered nucleases are protein-based tools, ZFNs and TALENs, used for the recognition of relatively long unique sequences in the genome (Figure 2).

The ZFN architecture consists of the *FokI* endonuclease cleavage domain from the *Flavobacterium okeanokoites* tethered to a tandem array of Cys_2_His_2_ type zinc-finger protein DNA-binding domains from a class of eukaryotic transcription factors connected using a short intervening amino acid linker [18]. The zinc-finger proteins, in the form X_3_-Cys-X_2-4_-Cys-X_12_-His-X_3-4_-His-X_4_ (X is any amino acid) folded into a ββα structure, each independently specifies three nucleotides [19]. Zinc-finger proteins can be designed to target a desired DNA sequence by linearly linking a combination of individual predetermined zinc-binding domains [20]. As a consequence of the *FokI* dimerization requirement to form a DSB, a pair of ZFNs is designed to bind to adjacent sites, 18 to 36 bp DNA sequence, on each strand in an inverted orientation and separated by a 5 to 6 bp spacer [21]. Generation of DSBs mediated by ZFNs is the precursor for different gene-editing outcomes that are facilitated by NHEJ or HDR [22,23]. Co-delivery of ZFNs along with donor template DNA in the form of single or double-stranded oligos or a viral vector with homologous arms can be used to mediate precise modification to correct a mutation or insert a new gene sequence. For example, ZFNs and donor templates in the form of oligos or integrase-defective lentiviral vectors (LVs) targeting the sickle cell mutation in the β-globin gene caused high levels of gene modification of up to 40% of alleles in human CD34^+^ HSPCs [24].

One major drawback of ZFN technology is the potential for unwanted gene editing at off-target sites that have mismatches to the target sequence that lead to toxicity in the form of cell death and apoptosis [25,26]. The cytotoxic effects of off-target gene editing by ZFN homodimers can be reduced using obligate heterodimers of ZFNs with *FokI* cleavage domains engineered to preferentially heterodimerize. However, the drawback of the obligate heterodimers is that they dimerize less efficiently than wild-type *FokI* cleavage domains, thus reducing cleavage activity [21]. Despite the reduced levels of on-target activity, the improved specificity of gene editing using these variants is particularly advantageous for increasing the safety of gene therapy applications. An additional limitation to use of ZFNs is their preference for GNN triplets [25]: the targeted site must be guanine rich, which reduces the range of targetable sites in the genome.

TALENs consist of the TALE DNA-binding domain from the *Xanthomonas* spp. proteobacteria tethered to the *FokI* catalytic domain as found in ZFNs [27]. The TALE-DNA binding domain is a modular array of 15.5–19.5 conserved repeats, each 33–35 amino acid residues in length. The individual TALE repeats bind to one nucleotide within the target sequence, which is determined by the repeat-variable di-residue (RVDs) at positions 12 and 13 within the module [28]. The formation of a DSB at the target locus is similar to the process in ZFNs: formation is mediated by a pair of TALENs that bind in a tail-to-tail orientation to two adjacent sites, with a total recognition sequence of 30 to 40 bp, separated by a 10–30 base spacer to facilitate *FokI* dimerization [29]. An advantage of TALENs over ZFNs is its broader targeting range: TALENs can be designed to target almost any DNA sequence in the genome because the RVD-DNA recognition code facilitates design of customized binding domains [30,31]. In addition, TALENs have cleavage activities comparable to those of ZFNs but are less cytotoxic; however, TALENs have the potential to induce mutations at off-target sites [32]. The unwanted off-target mutations generated by TALENs can be reduced or avoided by limiting the selection of the target sequence to sites having seven or more mismatches with any other genomic site, which reduces targetability when designing highly specific TALENs [33]. The advantage of ZFNs is that the cDNA encoding them, roughly 1 kb each, is significantly smaller than the 3 kb cargo for each TALEN; this makes delivery of ZFNs using viral vectors more feasible for gene therapy applications [34]. Furthermore, TALENs and ZFNs are severely limited by the inefficient, complex, and expensive process whereby they are assembled, making them impractical for academic laboratory settings.

**Figure 3:**
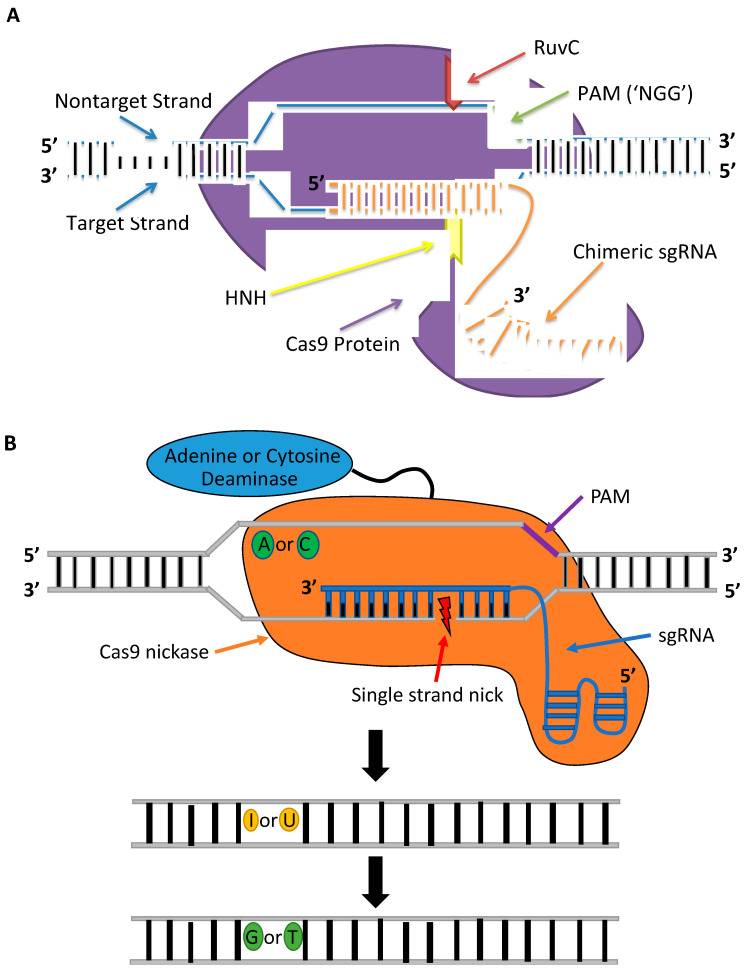
RNA-guided gene-editing tools. (**A**) An engineered 100 nucleotide sgRNA complexes with the Cas9 protein and directs it to a specific 20 nucleotide target sequence adjacent to the 5′ end of the PAM sequence. The 20 nucleotides of the sgRNA base-pair with the target strand, which positions the RuvC and HNH nuclease domains in the correct location to generate a DSB at the target site. (**B**) Mechanisms of Cas9 base editors for gene editing. The Cas9 nickase fused to a deaminase, deaminates a targeted adenosine (A) or cytosine (C) base converting it to inosine (I) or uracil (U). DNA polymerases read I as G and U as T and introduce the desired base pair to the sequence. This figure has been adapted from [6].

CRISPR-Cas constitutes a class of RNA-guided programmable nucleases. CRISPR-Cas is the adaptive immune system in most bacteria and archaea species that serves as a record of resistance to invading phages and plasmids that can be transferred to progeny [35]. The first RNA-guided system engineered and harnessed as a tool for genome editing in mammalian cells is the type II CRISPR system of *Streptococcus pyogenes* (Figure 3A), which is made up of a Cas9 (SpCas9) nuclease and guide RNA [36,37]. The guide RNA has the form of a dual-RNA complex, consisting of a target-specific CRISPR RNA (crRNA) and universal trans-activating CRISPR RNA (tracrRNA), or chimeric, single-guide RNA (sgRNA) with a 20 bp protospacer complementary to the target sequence. The guide RNA directs the Cas9 nuclease to the target site in the DNA where it activates a DSB 3–4 bp upstream a 5′-NGG protospacer adjacent motif (PAM) [38]. The guide sequence is highly tailorable and can be replaced to specify new target sequences whereas the remainder of the gRNA scaffold remains unchanged and so can target nearly any sequence in the genome that proceeds the PAM sequence. The simple design of CRISPR-Cas9 and the commercial availability of CRISPR reagents, including guide RNAs that can be rapidly synthesized in as little as 3 days, have facilitated its widespread use. In contrast to TALENs and ZFNs, CRISPR-Cas9 can be generated and validated more quickly and provide higher cleavage activities when delivered as ribonucleoprotein (RNP) complexes into mammalian cells [39]. The CRISPR-Cas9 system introduced as RNPs is less cytotoxic than plasmid DNA or mRNA. Plasmid DNA can be inserted at Cas9 cleavage sites, and mRNA can trigger a heavy antiviral interferon response that causes global transcriptional repression [39,40,41]. Furthermore, RNPs have a shorter half-life, disappearing within 48 h and causing lower off-target cleavage [42]. An additional advantage of the CRISPR-Cas9 system over protein-based nucleases is its multiplex gene-editing capability within the same genome [37]. These benefits have enabled CRISPR nucleases to become the gene-editing platform of choice for therapeutic applications.

One major disadvantage of the CRISPR-Cas system is its high levels of off-target gene editing at unintended sites that have up to five mismatches to the target sequence [43,44]. The off-target gene editing can have potential deleterious effects, namely chromosomal deletions and translocations, disruption of tumor suppressor genes or other essential genes, and activation of oncogenes [45,46]. These off-target cleavage events are a consequence of tolerated mismatches between the gRNA-DNA heteroduplex and DNA/RNA bulges downstream of the PAM; thus, it is necessary to thoroughly screen gRNA designs before their therapeutic application [47]. There are multiple in silico tools available for predicting putative off-target sites. The recently developed CRISPRitz platform is the only bioinformatics tool that simultaneously accounts for mismatches, bulges, and genetic variants while distinguishing between coding and noncoding off-target sites [48]. However, in silico CRISPR design tools cannot replace the need for additional validation of off-target gene editing using targeted deep sequencing or, ideally, whole-genome sequencing to accurately quantify the Cas9 specificity. Dual strand targeting using Cas9 nickases [49] and fCas9 [50], consisting of catalytically inactive dCas9 tethered to *FokI,* to form a staggered DSB have been shown to enhance the specificity of CRISPR-mediated gene editing without abolishing the on-target cleavage activity. However, these systems involve a pair of monomers and are complex and more difficult to design than Cas9 nucleases because two sgRNAs are required to bind adjacent sites, and for fCas9, simultaneously have sufficient spacing for *FokI* dimerization to initiate cleavage in both strands. The CRISPR-Cas9 system in which only one gRNA specifies the target site and minimizes off-target gene editing involves engineered Cas9 mutants. Multiple different SpCas9 variants have been shown to reduce off-target gene editing without compromising on-target activity in eukaryotic cells, including HypaCas9 [51], evoCas9 [52], and Sniper-Cas9 [53]. Nevertheless, not all high-fidelity Cas9 mutants that provide enhanced specificity when expressed by plasmids are as effective at on-target cleavage when delivered as RNPs. In the study by Vakulskas et al., the delivery of the high-fidelity SpCas9 variant (HiFi Cas9) as an RNP reduced off-target activity compared to the wildtype Cas9 RNP while maintaining robust on-target gene editing in CD34^+^ HSPCs [54].

A second major challenge associated with the CRISPR-Cas9 system is its large cargo size (~4.3 kb), which affects its delivery as a viral vector for gene therapy applications. This challenge is the impetus that led to characterization of Cas homologs from other species of bacteria. The CRISPR-Cas9 from *Staphylococcus aureus* (SaCas9) uses an sgRNA to bind to sites upstream of a 5′-NNGRRT PAM sequence. The SaCas9 has been employed in preclinical studies using animal models of human diseases that demonstrated successful therapeutic application [55,56]. In addition, SaCas9 reagents, similar to other Cas homologs, are commercially available to enable ease of their direct delivery as preformed sgRNA-Cas9 RNPs for potent cleavage activity at endogenous sites in cells. In contrast to SpCas9, the advantages of SaCas9 nuclease are its compact cargo size (~3.3 kb) and its multiple-turnover enzymatic activity, which has a faster rate of substrate release that causes no additional detectable nuclease activity to occur upon DNA cleavage [57]. The drawback of the SaCas9 is its limited targeting range due to its relatively long PAM sequence requirement. Luan et al. developed SaCas9 mutants, SaCas9-NR, and SaCas9-RL, which have a broader targeting range and improved activity than the wildtype SaCas9 [58]. The SaCas9 variants with relaxed PAM requirements have great potential for therapeutic application.

Base editors are catalytically dead or impaired CRISPR-Cas nucleases capable of introducing point mutations within a narrow region of a target site (Figure 3B) without performing DSBs or use of a donor template DNA [59]. Because base editors enable gene editing without activation of DSBs, base editors are useful tools for avoiding chromosomal deletions or rearrangements [59,60]. Base editors consist of an impaired Cas nuclease fused to a single-strand DNA deaminase enzyme and are divided into two main classes: cytosine [59] and adenine base editors [61,62]. Cytosine base editors make C-to-T conversions, while adenine base editors introduce A-to-G conversions. Recently, a third class of base editors containing a cytosine deaminase and an adenosine deaminase was shown to perform C-to-T and A-to-G conversions simultaneously [63]. Given that two-thirds of genetic diseases are caused by single point mutations [64], base editors are promising tools for therapeutic applications as demonstrated in preclinical studies using mouse models of human diseases [65,66,67].

In early studies, base editors showed low on-target gene-editing rates due to disruptions by native cellular repair mechanisms in cells. The creation of a mismatched base pair in the intermediate product could potentially activate the base excision repair (BER) mechanism. The BER pathway recognizes mismatches and performs a “base-flip” to correct the mismatch [68]. The first base editor system (BE1) was shown to perform efficient cytosine conversions in a test tube, but efficiency dropped drastically in human cell lines because of interference by the BER pathway [59]. The authors of the study suggested that the reduction in editing efficiency was due to BER of U•G mismatches induced by the base editors. Once a U•G mismatch is recognized, DNA uracil *N*-glycosylase (UNG) activates the repair mechanism and converts the U•G mismatch back to C•G. The authors suggested that inhibition of UNG could repress BER of U•G mismatches. The second generation of base editors (BE2) was designed to overcome the BER pathway by the addition of a uracil DNA glycosylase inhibitor (UGI) to the C-terminus of the base editor [59]. BE2 provided a three-fold increase in editing efficiency compared to BE1 while maintaining a low indel formation rate of ≤0.1%.

A major limitation of base editors is their high level of unintended nucleotide alterations, including indels or base pair conversions at the target site. To improve the activity of BE2, the dCas9 was replaced with a Cas9 nickase to generate BE3 [59]. The BE3 base editor provides higher base conversion activity compared to BE2 but also generates higher indels rates [59]. BE3 was introduced into mouse embryos by microinjection of mRNA encoding BE3 and sgRNA targeting *Dmd* and *Tyr.* While the most common substitutions observed in blastocysts grown from the treated embryos were the intended C-to-T, a sizeable number of blastocysts (20–36%) were found to also have unwanted C-to-A and C-to-G conversions [69]. Efforts to further limit unwanted products of base editing led to modifying the BE3 by fusing a second UGI to the C-terminus of the base editor construct to reduce inhibition by BER pathways and thus generate the BE4 system. BE4 was further improved by the addition of a Mu-derived Gam protein that binds to DSBs and limits indel formation [60]. A separate study also found that increasing expression of UGI in cells treated with BE3 reduced indels while increasing C-to-T conversions [70].

Off-target editing is a second major limitation of base editor technologies [69,70,71,72]. Adenosine deaminase base editors have been found to induce off-target editing, but at lower levels than cytosine base editors [73,74,75,76]. Digenome sequencing of HEK293 cells treated with BE3 showed that BE3 was more specific than SpCas9 [77]. The authors noted that, although gene-editing specificity was higher for BE3 than SpCas9, base editing at frequencies of 1–5% was observed for BE3 at off-target sites. In a separate study, the off-target editing was evaluated for adenine base editor ABE7.10 and showed base editing frequencies ranging from 0.1–7.8% at off-target sites [73]. Multiple strategies have been explored for lowering off-target activity of base editors. In the study by Rees et al., four point mutations were introduced into the BE3 to reduce off-target activity by reducing its affinity for DNA [71]. These mutations had been previously introduced into SpCas9 to generate the HiFi Cas9 that showed reduced off-target gene editing [78]: off-target editing was significantly reduced, and the on-target base editing activity was slightly altered. In addition, the study found that transient expression of BE3 by RNP delivery significantly reduced off-target editing compared to plasmid DNA. In a separate study, the rat APOBEC1 deaminase in the BE3 was replaced with human APOBEC3A cytidine deaminase, which significantly reduced off-target editing and unwanted cytosine conversions at the target site [79]. Extending the size of the sgRNA was shown to lower off-target activity without sacrificing on-target activity whereas truncated sgRNAs demonstrated decreased on-target editing and lower specificity than the extended sgRNAs. Although base editors have been shown to provide detectable gene-editing activity, future research should aim to improve the rate of on-target base editing while minimizing off-target editing and unwanted byproducts.

## 4. Delivery Strategies for Therapeutic Applications

### 4.1. Viral Systems

Viral delivery vectors have been used for the delivery of nucleic acids into cells and are the most-used delivery method for CRISPR-Cas9 due to the availability of well-established protocols and high transduction efficiencies. A major advantage of viral delivery systems is that having mechanisms for introducing genetic material into cells systemically, they facilitate in vivo delivery of gene-editing tools but have been engineered for safety by removal of the genes required for viral replication. Although there are numerous types of viral delivery systems, we will focus our discussion on adenovirus (AdV), adeno-associated virus (AAV), and lentivirus (LV)-mediated delivery of gene-editing tools in the proceeding paragraphs.

#### 4.1.1. Adenoviral Vectors

AdV is a nonenveloped, linear double-stranded DNA virus associated with cold-like symptoms and several other diseases. The AdV remains episomal and is suitable for transduction in both quiescent and dividing cell types [80]. As a delivery vector for CRISPR-Cas9-based gene editing, nonintegration is desired to minimize unwanted and sustained editing in off-target sites. Although in certain applications, such as development of Cas9 animal models, transient transgene expression is not desired. The first generation of AdVs had ~7.5 kb carrying capacity, elicited an acute chronic immune response, and were associated with high toxicity, which can be fatal in humans [81]. In an effort to eliminate the risks of immunogenicity, the latest generations of AdV vectors have more of the viral genes removed, as in the helper-dependent AdV design [80]. These modified AdV vectors have an extended carrying capacity of up to ~30 kb and have reduced immune reactivity [82]. Because AdV is commonly encountered in daily life, pre-existing immunity is a major issue. The study by Wang et al. demonstrated AdV-mediated delivery of CRISPR-Cas9 for efficient editing in the *Pten*, a negative regulator of the PI3K-AKT pathway involved in nonalcoholic steatohepatitis, in mice over 4 months. However, AdV-associated immunotoxicity in the liver was observed in treated mice [83]. Similarly, the study by Stephens et al. used AdVs to deliver CRISPR-Cas9 and donor template DNA to insert mFIX cDNA to the *Rosa26* safe harbor site that resulted in long-term phenotypic correction of Hemophilia B bleeding diathesis. However, adaptive immune responses against the AdV were detected [84]. The recent study by Liu et al. targeted the CCR5 gene by AdV-CRISPR-Cas12a, from *Acidaminococcus sp.*, to provide resistance to HIV-1 infection in CD4^+^ T cells [85]. The advantages of using AdV for CRISPR-mediated gene editing are the ease of production, well-defined structure, high efficiencies of transduction in vivo, and large packaging capacity [86]. However, use of AdV as a delivery vehicle for CRISPR-Cas nucleases is currently limited to research settings and cannot be translated to the clinic because of their immunogenicity risks.

#### 4.1.2. Adeno-Associated Viral Vectors

Adeno-associated virus (AAV) is a nonenveloped, single-stranded DNA virus in the parvovirus family. It is a small virus with an ~4.8 kb genome consisting of rep and cap open reading frames flanked by two inverted terminal repeats. AAVs require another virus such as AdVs or herpes simplex virus to enter the lytic cycle for replication. In the absence of a helper virus, the AAV enters the lysogenic stage by integrating into a specific region on the human chromosome where it remains in latency. AAV vectors are advantageous because they can be used for transduction in many cell types. Serological studies have revealed different serotypes of AAVs with different traits and various tropisms; the serotypes require appropriate selection of AAV vectors for specific target tissues. The most established serotype, AAV2, has a broad tropism for different tissues, including liver, muscle, brain, and retina [87,88]. AAV2 vectors integrate preferentially into the *AAVS1* on chromosome 19 with approximately 40% to 70% frequency [89]. Although the prevalence rate of AAV infection is high in humans (~60%), none of the serotypes has been linked to a known human disease [90]. Additionally, AAVs elicit only mild toxicity and low immunogenicity, indicating a stronger safety profile than that of AdV or LV vectors [80]. Removing more of the viral genes has yielded safer recombinant AAV (rAAV) vectors lacking the rep and cap viral genes that cannot integrate into the host genome. The rAAV viral genome forms circular concatemers through double-strand synthesis and remains episomal for long periods of time [91]. In dividing cells, the AAV episomes become diluted with each cell replication whereas in nondividing cells, the AAVs persist; this causes continuous expression. In pediatric patients, dilution of AAVs can cause loss of therapeutic transgenes as the patient’s tissues grow. In addition, in pediatric patients, rAAV integration is not completely eliminated and occurs with a 0.1% frequency in nonhomologous sites in the host genome [92].

i. Packaging CRISPR-Cas Components into AAV Vectors

One major limitation of the AAV vector is its small packaging capacity. SpCas9 is 4.2 kb, and the AAV system provides only 0.3 kb to package the sgRNA and other gene-editing elements. Senis et al. designed a single AAV vector using CMV, with a short polyadenylation signal, and H1 promoters for expressing Cas9 and sgRNA [93]. However, the limitation of the single AAV vector is that it cannot accommodate additional control elements for tissue specific expression or donor template DNA for HDR-mediated gene targeting. One solution to the carrying capacity issue is to use smaller Cas9 orthologs. St1Cas9 (3.3 kb) from *Streptococcus thermophilus* has been considered for this purpose although its PAM sequence narrows the range of possible targets [94]. SaCas9 has a 3.2 kb size, which enables a packaging capacity of ~1.3 kb for sgRNA and other elements. Moreover, editing efficiencies for SpCas9 and SaCas9 are similar, making SaCas9 an alternative for therapeutic applications [95]. The smallest Cas9 ortholog characterized to date is from the CRISPR system in *Campylobacter jejuni* (CjCas9). The ortholog has a 2.95 kb size for potential delivery of reporter genes and donor templates and multiple sgRNAs for multiplex editing from a single vector. The study by Kim et al. on treatment of age-related macular degeneration by targeting the *Hif1a* reported high specificity and comparable editing efficiencies for CjCas9 and SaCas9 when delivered in a single AAV vector in vivo [96]. A 1-year follow-up study showed persistent indels and no off-target editing [97]. In a separate study by Ibraheim et al., an all-in-one rAAV containing sgRNA and Cas9 ortholog from *Neisseria meningitidis* (3.16 kb) was used to disrupt *Pcsk9* in vivo, leading to lower cholesterol levels in mice. Results demonstrated successful gene modification with an efficiency of 35% at two weeks after injection [98]. Not all smaller Cas9 orthologs provide enough packaging space for the donor template DNA on a single vector. For example, a study by Yang et al. utilized two separate AAV vectors to incorporate SaCas9 driven under the TBG promoter, U6-sgRNA, and a donor DNA sequence to correct the mutation; this caused urea cycle disorder in the gene encoding ornithine transcarbamylase [99]. The dual AAV system for the delivery of Cas9 and sgRNA along with donor template is the most common strategy for CRISPR-Cas9 mediated gene targeting.

ii. AAV-Mediated Delivery for Therapeutic Applications

Many preclinical studies have reported successful gene editing using AAV-mediated delivery of CRISPR-Cas9 for treatment of a wide array of diseases, including hemophilia B [100], phenylketonuria [101], ornithine transcarbamylase deficiency [102], Huntington’s disease [103], amyotrophic lateral sclerosis [104], and familial hypercholesterolemia [105]. The retina is one of the most targeted sites for in vivo delivery of AAV-CRISPR-Cas9 because of its accessibility and immunologic privilege, which allows it to tolerate the introduction of antigens without elicitation of an inflammatory immune response. In these studies, the AAV vector-mediated CRISPR system was used to disrupt the disease-causing gene to prevent age-related macular degeneration [106], retinal angiogenesis [107], and retinal degeneration [108] with promising results. In a study using AAV delivery for CRISPR-Cas9, the IVS26 intronic mutation in the *CEP290* that causes Leber congenital amaurosis-10 was removed in a large deletion [109], demonstrating the applicability of the therapeutic approach for large gene deletions. In a separate study, congenital amaurosis-10 was corrected using CRISPR-Cas9-mediated targeting of the disease-associated nonsense mutation in *Rpe65* [110]. Furthermore, the study by Nishiguchi et al. used AAV-mediated delivery of CRISPR-Cas9 and a donor template to correct a mutation causing retinal dystrophy in 10% of photoreceptors with improvement in light sensitivity and enhanced visual acuity [111].

AAV CRISPR editing has been used to treat muscle disorders, such as Duchenne muscular dystrophy (DMD) [105,112,113]. In a recent study by Zhang et.al., Cas9 nuclease is packaged into a single-stranded AAV (ssAAV) and the sgRNAs into a self-complementary AAV (scAAV) to lower the dose by 20-fold for scAAV compared to ssAAV [114]. This efficiency difference stems from the ability of scAAV to reduce the lag time required to synthesize the second strand for expression. However, it should be noted that scAAV further reduces packaging capacity by 50% [89].

Despite systemic AAV delivery’s safety profile and track record in clinical trials, associated immunogenicity risks remain to be resolved. One major concern stems from pre-existing immunity against AAV capsids due to prior exposure to the wild-type virus. The first evidence of a CD8^+^ T cell response and pre-existing immunity was reported in a clinical gene therapy trial for severe hemophilia B involving rAAV delivery of *FIX*. At 8 weeks after injection, all patients had therapeutic FIX levels, transient increase in liver transaminase levels, and elevated neutralizing antibody titer accompanied by subsequent decrease in transgene expression to baseline [115]. In a study by Jiang et al., pre-existing immunity and consequent increase in neutralizing antibody levels were shown to result in a complete block of transduction when AAVs were injected at a high dose [116], suggesting that inhibition of AAVs by neutralizing antibodies was dose-dependent. Additional studies confirmed AAV-specific immune responses in both animal models [117,118,119] and humans [120,121,122] and showed that low titers of neutralizing antibodies can avert AAV transduction [115]. Notably, it was reported that as the dose of neutralizing antibodies increased, the biodistribution pattern of AAV transduction changed to increased targeting of immune organs such as the spleen [119]. Prior to being selected to enroll in AAV-based clinical trials, patients are subjected to screening using neutralizing assays to detect pre-existing immunity. However, considering the high prevalence of AAV infection in humans for all serotypes [123] and the long term persistence of immunity [124], excluding the population with preexisting immunity from AAV treatment is considered not ideal. Switching to different serotypes and using high vector doses have been proposed to overcome humoral immunity, but the high cross-reactivity of neutralizing antibodies and toxicity of high doses restricts these strategies. Moreover, chimeric forms of AAVs engineered to combine different serotypes have been explored to reduce immunogenicity issues. In a phase 1 clinical gene therapy study by Bowles et al., a chimeric AAV capsid variant, AAV2.5, derived from AAV2 and AAV1 serotypes was shown to partially escape humoral immunity with ~2–5.5-fold higher transgene expression and ~2–20 fold lower neutralizing antibody titer when injected at high doses [125]. However, high doses constitute a risk of toxicity [124], and it is not known whether the same effects would be observed with lower doses. Plasmapheresis has been explored: The blood of the patient is withdrawn to remove large molecular weight molecules such as antibodies from the plasma, and immunosuppressants, which are associated with safety concerns, are administered [126].

The integration frequency of rAAV is very low; nevertheless, the genotoxicity risks still constitute valid safety concerns in the context of therapeutics, since 0.1% integration translates to a considerable number of integration events. The random integration of AAVs has the potential to result in mutagenesis due to incorporation of the vector into or near cancer-driver genes. In fact, evidence from two studies revealed that insertional mutagenesis to *Rian* locus after systemic administration of AAV vectors in neonatal mice caused hepatocellular carcinoma [127,128]. The findings led to controversy: separate studies reported no AAV integration events [129]. Moreover, it was argued that the *Rian* locus is not conserved in humans, suggesting that mutagenesis is species-specific. However, findings of clonal integration sequences from AAV2 at known cancer driver genes, including *CCNA2*, *CCNE1*, *KMT2B,* and *TERT* in liver samples further raised safety concerns [130]. A recent study reinforced the role of AAV insertional mutagenesis in hepatocellular carcinoma with reports of clonal AAV insertions in various cancer-driver genes in 2% of treated patients [124]. The accumulating evidence highlights the need for more investigation on rAAV integration with comparative studies.

Although most of the AAV-based CRISPR applications focus on in vivo genome editing, ex vivo editing provides an alternative approach for gene editing that avoids the safety concerns associated with systemic delivery of AAVs. Dever et al. reported successful ex vivo gene editing using the AAV-based CRISPR system targeting the *HBB* gene in CD34^+^ HSPCs to correct the mutation causing sickle cell disease [41]. Recently, a single AAV vector containing SaCas9, gRNA, and donor repair template was used to correct the mutation in *Fah* that causes hereditary tyrosinemia-1 ex vivo in hepatocytes [131]. The study demonstrated successful repopulation of the liver by gene-modified hepatocytes, resulting in a complete rescue in mice without the detection of abnormalities.

#### 4.1.3. Lentiviral Vectors

Lentiviruses (LV) belong to the *Retroviridae* family, which carries genomic material as RNA and has the capacity to retrotranscribe RNA into DNA using reverse transcription. After the virion binds to its receptor on a target cell, it enters the cell by endocytosis or fusion and releases its core, including reverse transcriptase and integrase. With the help of host nucleotides, reverse transcriptase synthesizes cDNA from the viral RNA and subsequently transports the cDNA to the nucleus where it integrates into the host genome [132]. Although both lentivirus and γ- retrovirus belong to the same family, lentiviruses are complex retroviruses expressing accessory genes in addition to gag, pol, and env genes [133]. LV vectors have ~8 kb carrying capacity, and unlike simple retroviruses, they can infect both dividing and nondividing cells due to their unique ability to actively transport through the nuclear pore of an intact nuclear envelope [134]. LVs also have a broad tropism. Moreover, the large cargo capacity allows for simultaneous delivery of both Cas9 and sgRNA using a single LV vector, providing an advantage over AAVs. Additional advantages of LV vectors include high efficiency of gene editing in a wide variety of cell types and low immunogenicity in humans [135,136,137].

Because of the life cycle of retroviruses, LVs integrate into the host genome. Viral integration may be desirable for applications where long-term transgene expression is required, such as when making gene libraries or model organisms. However, for therapeutic approaches, LVs carry safety risks for insertional mutagenesis as well as persistent expression of site-specific nucleases leading to off-target mutations [132]. The first-generation lentiviral packaging system contains a significant portion of the HIV genome [138], which has the potential to undergo replication-competent virus generation. To address this concern, self-inactivating (SIN) lentiviral vectors have been developed by deleting a segment of the LTR to abolish the promoter activity. In second- and third-generation LV systems, the accessory genes of HIV were deleted, and a split-plasmid system was utilized to improve safety [139,140]. Alternatively, mutating the integrase gene has given rise to a nonintegrating lentivirus (NIL). Some studies show that NILs have reduced efficiency [141], with 2–10 fold lower expression in vitro compared to conventional LVs, yet separate studies show that NIL and conventional LV vectors have equivalent transduction efficiencies in vivo [139]. The integrase activity, nevertheless, was not completely eliminated, and a residual integration was reported [142]. Additional studies are needed to address these limitations. Given the safety risks, care must be taken with using LVs for therapeutic gene editing.

i. LV-Mediated Delivery for Therapeutic Applications

There have been many studies that use LVs for the delivery of CRISPR components for therapeutic applications and show promising results. The study by Wang et al. used the CRISPR-Cas9 system to disrupt the CCR5 gene to make cells resistant to HIV-1 infection using LV-mediated delivery. One round of co-transduction was sufficient to obtain a high frequency of *CCR5* disruption without any off-target mutations, and the transduced cells exhibited resistance to infection by R5-tropic HIV-1 and a selective advantage over cells carrying wild-type CCR5 [143]. A similar study by Hou et al. demonstrated successful disruption of *CXCR4* using LVs containing CRISPR-Cas9 in human CD4^+^ T cells [144]. The gene-modified T cells showed resistance to X4 tropic HIV-1 infection without any off-target mutations [145]. The study by Yu et al. combined both approaches, while demonstrating simultaneous knockout of *CXCR4* and *CCR5* using an LV containing Cas9 and sgRNAs to obtain resistance to both R5- and X4-tropic HIV-1 infection in susceptible cell lines without any off-target mutations. Nonetheless, the rate of edited cells homozygous for both genes was relatively low, at ~10% [146]. Roehm et al. demonstrated LV-mediated delivery of CRISPR-Cas9 to introduce disrupting indel mutations in the *ICP0* to prevent Herpes simplex virus type 1 (HSV-1) infection. The study showed successful abrogation of HSV-1 infection in uninfected cells by blocking viral replication and a drastic reduction of HSV-1 replication in the infected cells [147].

Studies have explored the use of CRISPR-Cas13, which targets RNA, to combat retroviral infections. Cui et al. adapted CRISPR-Cas13b to directly disrupt the viral RNA of porcine reproductive and respiratory syndrome virus (PRRSV) in eukaryotic cells. They delivered Cas13b and crRNAs targeting the PRRSV essential genes *ORF5* and *ORF7* through LV transduction and obtained an almost complete loss of genomic RNA when targeting simultaneously [148]. More recently, Abbott et al. used LV-mediated CRISPR-Cas13-based strategy to provide a prophylactic antiviral tool for the SARS-CoV-2 [149]. LV was used to deliver CRISPR components for noninfectious diseases. The study by Holmgaard et al. used LVs containing sgRNA and SpCas9 targeting *Vegfa*, the gene whose dysfunction is associated with age-related macular degeneration. The CRISPR-Cas9 mediated knockdown of genomic *Vegfa* was observed after subretinal injection in mice caused editing efficiencies up to 93% and 84% in vitro and in vivo, respectively [150].

An all-in-one LV vector was used to deliver gRNA-dCas9-DMNT3A (DNA methyltransferase-3) targeting CpG islands in *SNCA* intron 1 to obtain an indirect downregulation of SNCA expression through DNA methylation [151]. The objective of the study was to harness an epigenetic editing strategy for the manipulation and control of SNCA transcription, which is implicated as a genetic risk factor for Parkinson’s Disease (PD). When applied in human induced pluripotent stem cell-derived neurons from a PD patient, the CRISPR system resulted in hypermethylation of the triplicated *SNCA* locus leading to downregulation of expression, causing the rescue of disease-related phenotypic perturbations, including cell viability, mitochondrial function, and oxidative stress. Importantly, significant off-target DNA methylation was observed in the control group harboring no gRNA, indicating a need to improve the specificity and the safety of the system.

ii. LV-Mediated Delivery for Genomic Screenings

LVs are widely used for the creation of gene libraries and genomic screening. Shang et al. used LVs to perform a genome-wide CRISPR screen in search of genes that regulate T cell activation and successfully identified a previously uncharacterized regulator, FAM49B [152]. Genome-wide knockout screening strategy utilized LVs to pack large libraries of sgRNAs to make pools of cells with diverse genomic modifications [153]. The study by Shalem et al. successfully demonstrated the feasibility of conducting genome-scale CRISPR-Cas9 knockout screening with a pooled LV library to deliver Cas9, sgRNA, and a selection marker. The authors designed a library of 64,751 sgRNAs targeting 18,080 genes in the human genome that enables both negative and positive selection screening in human cells. They observed a high efficiency of complete knockouts and successfully found candidate genes with high validation rates [64]. Han et al. used the same strategy to identify host factors essential for influenza virus replication and identified capicua as a negative regulator of cell-intrinsic immunity [154]. The discovery of the host factors and receptor for Norovirus, the leading cause of gastroenteritis, was the result of CRISPR screens using LVs for the delivery of the sgRNA library [155]. Similarly, Sun et al. identified new drug targets conferring sorafenib resistance in the treatment of hepatocellular carcinoma [156].

### 4.2. Electroporation

Electroporation is a physical transfection method that applies high-voltage currents to cells to permeabilize the membranes to nucleic acids, chemicals, or proteins. After the application of an electrical pulse, small gaps open up in the cell membranes, allowing the entry of substances; these gaps subsequently reseal. Electroporation-mediated gene therapy was first demonstrated in 1982 in delivery of plasmid DNA into mouse L cells deficient in thymidine kinase [157]. Initial in vitro studies established that electroporation is efficient at delivering Cas9 into cells considered difficult to transfect, such as primary fibroblasts [39], human embryonic stem cells [39], pluripotent stem cells [158], and neurons [159]. Therapeutic applications of electroporation for gene editing are largely limited to ex vivo strategies. Recently, however, electroporated-mediated delivery of Cas9 has progressed to clinical trials [160,161].

Electroporation has been extensively used for delivery of Cas9 into CD34^+^ HSPCs for treatment of genetic and acquired diseases [41,162,163]. This gene-editing strategy involves isolating CD34^+^ HSPCs from patients, gene modifying the cells ex vivo, and infusing them back into the patient for the treatment of HIV, sickle cell anemia, and β-thalassemia. Strategies for therapeutic gene editing to treat HIV typically attempt to disrupt the HIV co-receptor *CCR5* [164]. Early in vitro experiments demonstrated successful gene targeting in *CCR5* by electroporation of Cas9 and single sgRNA into cell lines [43,158]. However, in HSPCs, delivery of Cas9 along with a dual-targeting sgRNA system was necessary to disrupt *CCR5* [162]. A preclinical study used electroporation and a dual sgRNA system to transfect human CD34^+^ HSPCs; these were then implanted into immunodeficient mice [53]. In treated mice, gene modified cells engrafted into the bone marrow, resulting in lower HIV levels compared to control mice and indicating that the ex vivo CRISPR treated HSPCs were HIV resistant. In a subsequent phase I clinical trial, Cas9 treated HSPCs with ablated *CCR5* were transplanted into a patient positive for HIV and in remission for acute lymphoblastic leukemia [160]. Although the gene-edited cells engrafted into the bone marrow, the level of *CCR5*-deficient cells was <10% and insufficient to confer HIV resistance to the patient. The low levels of gene modified HSPCs engrafted into the bone marrow are likely the result of poor cell functionality and viability after electroporation.

Therapeutic gene-editing strategies for sickle cell anemia and β-thalassemia either correct the mutated β-globin [165] or disrupt the repressor *BC11A* gene [42,166]. In vitro studies for delivery of β-globin-targeting Cas9 RNPs showed higher levels of gene editing and improved toxicity compared to mRNA [41,165] or lentiviral delivery [165] but resulted in low levels of HDR-mediated gene correction. In a preclinical trial that employed electroporation to deliver β-globin-targeting Cas9 RNPs into human HSPCs; the cells were subsequently xenografted into immunodeficient mice and showed stable engraftment over four months [167]. Treated mice showed an increase in wild-type hemoglobin, and corrected cells expanded in vivo following engraftment, indicating this strategy is viable for future clinical studies. However, initial rates of HDR were low (6–11%), and cells required expansion for 5 days to increase the levels of gene corrected cells. An alternative strategy involving nuclease-mediated knockdown of *BC11A* to increase production of γ-globin has been proposed for sickle cell anemia and β-thalassemia [168]. One study found that deletion of a 200 bp sequence in *BC11A* with Cas9 significantly increased fetal hemoglobin levels in vitro [169]. Currently, there are two clinical trials examining the feasibility of disrupting *BC11A* by electroporation-mediated delivery of Cas9 into HSPCs for the treatment of β-thalassemia and sickle cell disease (NCT03655678, NCT03745287) [170].

Electroporation-mediated therapeutic gene editing has been applied for T cell cancer immunotherapies. In vitro studies demonstrated successful gene editing in T cells electroporated with CRISPR-Cas9 for recognition of cancerous cells [171,172]. In an effort to create “universal” T cells, multiplexed Cas9 editing at three target sites has been investigated using electroporation for delivery in preclinical studies to decrease T cell immunogenicity and improve antitumor recognition [173,174]. A phase I clinical trial examining the feasibility of multiplex Cas9 engineering in T cells by electroporation showed enhanced antitumor targeting [161]. In this trial, three patients with refractory cancer were treated with Cas9-edited cells that persisted without toxicity for up to 9 months following treatment, indicating that multiplex gene editing of T cells is a safe procedure. However, sequencing of treated T cells showed that only ~10% of cells had mutations at all three target sites, and ~30% had no mutations at all. This indicates that electroporated-mediated delivery of Cas9 into T cells requires further optimization for multiplex Cas9 gene editing. 

Despite success in preclinical trials, advancements are needed to further enhance electroporation-mediated delivery of Cas9 for clinical trials. Low levels of gene knockout or knockin require screening of ex vivo treated cells to enrich for successfully gene edited cells prior to transplantation. In contrast, screening is unnecessary when there are high levels of transfection efficiency. Studies have found decreased viability in cells following electroporation [175]. Optimization of electroporation should focus on increasing transfection efficiency while minimizing cell toxicity. Nucleofection is a form of electroporation that delivers agents directly into the nucleus, which may improve Cas9 efficiency. Studies that have used nucleofector devices have observed gene-editing rates of >90% in treated cells [176]. Other studies have indicated that modifying the vessel design to ensure even voltage across the electroporation buffer can achieve high levels of editing (>70%) in neural stem cells and induced pluripotent stem cells while simultaneously lowering the effects of toxicity [177]. Another potential method for improving viability is to optimize the electroporation buffer. Studies have reported that the addition of Mg^2+^ ions to the buffer can improve viability although the transfection of DNA can be impaired at excess concentrations [178,179]. Further optimization of electroporation methods for delivery of Cas9 should be investigated to ease transition into clinical applications.

### 4.3. Lipid Nanoparticles 

Lipid mediated delivery of gene products into cells was first demonstrated in 1987 [180]. Cationic lipids are nanostructures that consist of a cationic head group, a hydrophobic tail, and a linker between these two domains [181]. Cationic lipid nanoparticles (LNPs) bind to negatively charged nucleic acids to carry them across the cellular membrane [37]. Encapsulation by a lipid layer provides protection from RNases and degradation enzymes [182]. Cationic LNPs have been investigated for delivery of siRNA [183] as well as mRNA [184] and have progressed to clinical trials [185,186,187,188]. However, no clinical trials are currently underway for LNP delivery of CRISPR-Cas9 systems. Further optimization is necessary for successful transfection. Current challenges with LNPs for Cas9 delivery are low rates of transfection [189,190] and targeting specific tissues [191].

Early experiments for lipid-mediated delivery of Cas9 often used Lipofectamine to deliver plasmid DNA expressing Cas9 and a targeting sgRNA into cells [192,193,194]. However, the transfection rate for Lipofectamine is typically low. To improve transfection efficiency, LNPs and a viral vector were combined for delivery of CRISPR-Cas9 and donor template DNA into hepatocytes in vivo in a mouse model of human hereditary tyrosinemia [190]. Despite improved disease symptoms in treated mice, gene correction was low, roughly 6% of hepatocytes. In addition, lack of target specificity and safety concerns due to possible nonspecific interaction of positively charged lipids with matrix components along with their cytotoxicity in large doses limits the therapeutic application of LNPs [195]. Attempts to improve the efficiency of Cas9 mRNA delivered into cells included use of zwitterionic amino lipids (ZALs) [189]. These nanoparticles were designed for the ~4500 nt Cas9 mRNA. The authors reported that small changes to the poly ethylene glycol (PEG) lipid ratio in ZAL lipids had significant effects on the efficiency of the Cas9 mRNA in vitro. As PEG ratios increased, mRNA efficiency decreased. However, in vivo delivery of ZALs showed low gene-editing activity of 3.5%. A similar study achieved higher gene-editing activity of 35% in mouse hepatocytes in vivo by injecting two doses of lipid-like nanoparticles containing Cas9 mRNA and sgRNAs targeting *Pcsk9* [196]. The first study to demonstrate high levels (>70%) of gene knockout in vivo delivered Cas9 mRNA with a biodegradable LNP and chemically modified sgRNAs [197]. The authors engineered an LNP with a helper lipid along with PEG-Dimyristoyl glycerol and delivered Cas9 targeting *Ttr* in mice. The group also compared Cas9 activity in vivo with unmodified sgRNAs and chemically modified sgRNAs. Lower levels of Cas9 editing were observed in vivo using unmodified sgRNA, indicating that chemically modified sgRNAs with enhanced stability are essential for in vivo editing.

Because the Cas9 protein has a positive net charge of +22, it does not readily bind and assemble into LNPs [198]. However, sgRNA has an anionic charge of −103 that results in the combined Cas9-sgRNA complex having an overall negative charge for binding to cationic LNPs [198]. In Zuris et al., high levels of gene disruption of ~80% were observed with the delivery of the sgRNA-Cas9 complex in vitro when using Lipofectamine 2000. However, using Lipofectamine 2000 was also associated with an increase in toxicity. In vivo delivery of Cas9-sgRNA to the cochlea of mice was shown to result in a gene-editing efficiency of ~20%. In addition, delivery of the Cas9-sgRNA complex reduced off-target editing compared to plasmid transfection. In Gao et al., a bioreducible lipid was used to deliver Cas9-sgRNA complexes into cultured human cells with efficiencies greater than 70%. Cas9 RNP complexes were delivered in vivo in neonatal mice with Lipofectamine 2000 to treat hearing loss in a mouse model [199]. Treated mice showed improvements in hearing compared to untreated control mice. However, sequencing found only low levels of editing at the target site in both primary cells and dissected tissue from treated mice. These studies indicate that delivery of Cas9 by cationic LNPs for therapeutic gene editing is promising but requires further optimization for clinical use.

Delivery of Cas9 to specific tissues remains an ongoing challenge for LNP-mediated CRISPR-Cas9 delivery. In a study by Wei et al., a wide array of LNPs was shown to be tunable for delivery of Cas9 into targeted tissues, including w dendrimer lipid nanoparticles, stable nucleic acid lipid particles, and lipid-like nanoparticles [191]. Injection of cationic LNPs with increasing amounts of a permanent cationic lipid, 1,2-dioleoyl-3-trimethylammonium-propane (DOTAP), showed that 60% DOTAP enhanced specific delivery of Cas9 to lung tissue while lower percentages than 5% resulted in delivery into the liver. The introduction of a tunable tissue-specific method for delivering LNPs opens potential therapeutic applications for lipid-mediated delivery of Cas9. In a separate study, an alternative strategy for tissue-targeted delivery of Cas9 was demonstrated using pH-sensitive liposomes [200]. The authors demonstrated that pH-sensitive liposomes disassembled in the presence of the tumor microenvironment, releasing the Cas9 protein and allowing for gene editing. Tunable LNPs represent a promising avenue for advancing clinical application of LNPs for in vivo gene editing.

### 4.4. Hydrodynamic Delivery

Hydrodynamic delivery (HD) is a physical delivery technique originally developed for in vivo introduction of naked DNA in 1999 [201,202]. Since then, the technique has been applied for delivery of other molecules, including siRNA and small chemicals [203,204]. During the procedure, large volumes of gene solution are injected rapidly into circulation to create a high pressure that results in the transient permeabilization of the cell membrane to enable intracellular gene transfer. In rodents, the tail vein is a convenient route for delivery as it is easily accessible.

Mechanistically, upon tail vein injection, the solution enters circulation through the inferior vena cava and flows through the heart, but due to the excessive load, retrograde flow occurs into the hepatic vein, where the solution is forced out into tissues [205]. As a result of this mechanism, the procedure is especially effective for liver-targeted deliveries. Tracking studies in mice using GFP revealed that the majority of exogenous material hydrodynamically injected into the tail vein accumulates in the liver with weaker signals from other organs, including spleen and pancreas [206,207,208]. To achieve delivery to nonliver tissues, the solution can be directly injected into the vessels supplying the desired organ, enabling more specific and targeted delivery. For example, successful HD targeting to the kidneys can be achieved by injection in the renal vein; to the brain through injection in the carotid artery; and to the myocardium through injection in the vena cava [205,208]. To target muscles using the HD approach, direct intramuscular injection or vascular injection of gene constructs through the hind limb vein is performed [205,209]. The HD approach is advantageous in terms of simplicity and cost effectiveness, and it enables in vivo delivery without the use of viral vectors.

i. HD for Therapeutic Applications

HD has been applied for delivery of CRISPR-Cas for in vivo genome editing in models of human inherited metabolic diseases affecting the liver. Yin et al. achieved successful in vivo gene correction of the mutation in *Fah*, the gene encoding fumarylacetoacetate hydrolase, in a mouse model of hereditary tyrosinemia by HD of CRISPR-Cas9 components along with a donor template DNA. The overall editing efficiency of 4% of the hepatocytes was sufficient to correct the disease because the edited hepatocytes had a selective advantage over the unmodified cells for repopulating the liver [13]. The Fah-deficient mouse model was similarly corrected in the study by Ibraheim et al. using a different gene-editing approach based on reprograming the tyrosine degradation pathway. In the study, the Nme CRISPR-Cas9 system, delivered by tail vein injection, introduced indels to disrupt *Hpd*, the gene encoding hydroxyphenylpyruvate dioxygenase, with indels within the range of 35–60% to prevent the accumulation of toxic metabolites in the liver [98]. More recently, the study by Song et al. demonstrated gene correction using the adenine base editor and *Fah*-aiming sgRNA to correct an A-G splice-site mutation that caused hereditary tyrosinemia type I in the liver of Fah-deficient mice hydrodynamically injected via the tail vein [210]. Although the efficiency of the adenine base editor was low (roughly 0.1%), the gene corrected hepatocytes proliferated in the liver, resulting in weight stabilization and protection from liver failure in treated mice. In a separate study, HD of CRISPR-Cas9 was demonstrated for treatment of fulminant hepatic failure [211]. In the study, the *Fas*, encoding for a death receptor associated with apoptosis, was disrupted using CRISPR-Cas9 to rescue Concanavalin-A-induced fulminant hepatic failure in a mouse model. Results demonstrated successful downregulation of Fas expression, leading to the protection of hepatocytes from Fas-mediated apoptosis.

In a separate study by Zhang et al., CRISPR-Cas9 introduced using HD was used to correct mouse models of Hemophilia A [212]. The authors delivered a donor template that would lead to the integration of B domain-deleted *FVIII* (BDDF8) into the *Alb* locus along with CRISPR components via hydrodynamic delivery and observed 0.1% and 2% knockin efficiencies with circular and linearized plasmid donors, respectively. Moreover, the linear donor design led to successful precise integration of *BDDF8* in 1–2% of liver cells and corrected hemophilia A in most of the affected mice.

CRISPR-Cas9 delivered via HD injection has been demonstrated for treatment of infectious diseases in mouse models. The study by Lin et al. was the first to demonstrate the efficacy of harnessing CRISPR-Cas to target the virus causing Hepatitis B (HBV) in vivo [213].Using hydrodynamic injections to deliver CRISPR-Cas9, the authors reported a cleavage efficiency of -5% within the intrahepatic HBV DNA at 6 days after injection with a reduction in, but not complete elimination of, the hepatitis B serum surface antigen (HBsAg) levels [214]. Zhen et al. used the same approach involving HD of CRISPR-Cas9 targeting the surface antigen (HBsAg)-encoding region of HBV in vivo and obtained up to 85% inhibition of HBV antigen expression with a synergistic effect when two sgRNAs were combined to make a deletion in the S1/X3 antigen/polymerase region in the provirus [215]. Although there are licensed treatments available that effectively suppress hepatitis B virus (HBV) replication, they cannot eliminate the replicative templates of the virus [216]. To investigate use of CRISPR-Cas9 on eliminating HBV DNA, Dong et al. used an HBV mouse model and reported efficient inhibition of HBV replication and decreased replicative HBV DNA in mice injected with sgRNA and Cas9 plasmids [217]. Using the same approach, but an SaCas9, Liu et al. obtained similar results of significantly lower HBV protein expression [218].

ii. HD for Generation of Animal Models of Human Disease

HD has also been utilized as a tool to study the molecular basis of diseases and create animal models. Engelholm et al. used HD of CRISPR-Cas9 designed to delete the syntenic region on chromosome 8 in mice. Successful deletion was observed in 80% of the mice and resulted in a *Dnajb1–Prkaca* fusion mutation, which has been identified in 80–100% of patients with fibrolamellar hepatocellular carcinoma (FL-HCC), a rare form of liver cancer whose molecular basis is not established [219]. The mutated mice demonstrated development of neoplasms and had histologic and cytologic features of human FL-HCCs that further confirmed the *Dnajb1–Prkaca* fusion mutation as the cause of FL-HCC in wild-type mice. The study not only brings to light the molecular basis of the disease by identifying causal mutations, it provides a potential therapeutic target while establishing a mouse model of FL-HCC.

In a separate study, Gao and Liu investigated the role of cross-talk between Pten loss and Nras activation in driving liver cancer development in mice [220]. Using immunocompetent CD-1 mice, the authors delivered plasmids carrying Sleeping Beauty transposon-based integration of *Nras* and the *Pten*-aiming CRISPR-Cas9 through hydrodynamic injection. The results showed that only the combination of *Pten* disruption and Nras overexpression (but neither alone) was sufficient to induce HCC development in mice. By revealing the crosstalk between these two genes, the findings provide insight into HCC development, which may have important implications in liver cancer research.

Although HD has proven to be useful in small animal models, major safety concerns prevent its translation to the clinic. One major hurdle is that the injected gene solution comprises roughly 10% of bodyweight [201], which is feasible in small animals, such as rodents. In humans, the equivalent would translate to injecting 6 L of solution for a 60 kg person, which is not applicable clinically. To improve the safety of HD injection in humans, research has been focused on decreasing the required volume of gene solutions. One approach is to employ a target-specific injection using catheters instead of systemic delivery. Yokoo et al. described a liver-targeting procedure that involves a catheter insertion to each hepatic lobular vein for a temporary occlusion of flow; this causes a desired intravascular pressure without use of extreme volumes of solution [221]. Using this method, the volume of solution injected was reduced to <1% of body weight for each liver lobe in a swine model [222]. Moreover, a computer-controlled delivery system was developed and combined with the catheter method, which allowed for real-time control over flow and pressures [223,224]. Although the reduced volume is safer, the level of gene expression achieved is significantly lower than the conventional procedure of hydrodynamic injection in rodents [209,222].

Another major drawback of HD is the adverse effect of the injection. Upon injecting 9% of body weight into mice, a sharp increase in intravascular pressure in the liver and hepatocellular vascularity along with morphological changes were observed [225,226]. The temporary occlusion of the portal vein was followed by an elevation in liver biochemical markers [225,227], indicating poor liver health. Moreover, hemodynamic changes have been observed to cause immediate cardiac dysfunction due to transient cardiac overload in some animals including rats [228,229]. Although in small animals adverse effects from HD are quickly reversible without long-term complications [227,228], these effects preclude the use of HD for clinical applications.

### 4.5. Cell-Penetrating Peptides

A potential tool for direct delivery of Cas9 into cells is to fuse the nuclease to a cell-penetrating peptide (CPP) that carries cargo across a cellular membrane. CPPs were first described in 1988 when researchers observed that the transcription transactivating protein isolated from the HIV virus could cross cellular membranes [230]. For delivery of gene-editing tools, CPPs have been observed to successfully introduce ZFNs [231], TALENs [194], and Cas9 [232] in cells in vitro. For CRISPR-Cas9 delivery, two separate CPPs were used for the Cas9 protein and sgRNA, resulting in low levels of gene editing in HEK293T, HeLa, NCCIT, human fibroblasts, and embryonic stem cell lines. In a separate study, amphiphilic penetrating peptides were synthesized by fusing a cationic peptide with a hydrophobic aldehyde tail with a hydrazone bond for delivery of the Cas9 RNP [233]. In HeLa cells, the modified CPP had cleavage activity comparable to that of Cas9 delivered using Lipofectamine 2000. In addition, the authors confirmed that the modified CPP was effective in other cell lines, such as human lung epithelial A549 and the chicken fibroblast DF1. Cells treated with the modified CPP were also shown to have lower toxicity when compared to Lipofectamine 2000. CPPs have potential for Cas9 delivery into cells for therapeutic gene editing; however, few studies have examined CPP-mediated delivery in vivo.

## 5. Conclusions

The development of gene-editing technologies has occurred at a rapid pace with new tools or variants of existing tools emerging almost every year. In light of gene-editing-based gene therapy trials well underway in the US, Europe, and China, modern medicine has embarked on a quest in which most monogenetic and acquired diseases have the potential to be treated or cured at the genomic level by precisely altering sequences at specific loci in human cells. As the most used and characterized gene-editing tool, CRISPR-Cas9 nuclease is currently the platform of choice for emerging translational applications of gene editing. A major barrier to clinical advancement of Cas9-mediated gene-editing therapies is the absence of safe and effective methods for delivering the CRISPR components into target cells. AAVs are a practical delivery approach for introducing CRISPR-Cas9 reagents, particularly the SaCas9, a small ortholog amenable to packaging into a single AAV vector for in vivo gene editing. However, AAVs have the potential to randomly insert into the genome, and they increase risk of hepatocellular carcinoma in neonatal mice [127,128,220]. Furthermore, AAVs are associated with cell-mediated and pre-existing immunity [115,234,235,236]. Because AAVs exist as stable episomes, there are concerns that persistent Cas9 expression will occur, potentially causing increased off-target activity and genotoxicity [237]. An additional barrier for the therapeutic application of CRISPR-Cas is the substantial prevalence of pre-existing Cas9 immunity in the human population, with up to 78% of individuals having anti-Cas9 IgG antibodies and Cas9-specific T cells [238,239]. In the study by Li et al., AAV containing CRISPR-Cas9 introduced into a host with pre-existing immunity led to cytotoxic T cell responses and elimination of gene-modified target cells in vivo [55], providing evidence that Cas9 immunity cannot be circumvented by AAVs.

Non-viral methods have the potential to address the challenges of AAV-mediated delivery for introducing gene-editing reagents into target cells. LNP-mediated delivery of transient Cas9 mRNA or RNPs is a potential solution to pre-existing immunity. However, because Cas9 mRNA and RNPs can provide peak gene editing for up to 48 h after their introduction in cells [39,42], there remains a possibility that cells may present Cas9 peptides on the major histocompatibility complex class I surface proteins that can trigger cytotoxic T cells. In contrast, ex vivo Cas9-mediated gene editing is potentially safer than in vivo approaches because the engineered target cells can be maintained in culture until Cas9-derived peptides are no longer expressed on the major histocompatibility complex class I proteins on the cell membrane prior to infusion back into the host. However, studies are needed to define the culturing time to eliminate Cas9 proteins on the major histocompatibility complex I molecules. In addition, separate studies show Cas9 triggers a toxic P53-mediated DNA damage response that inhibits gene editing but has a protective mechanism to eliminate aberrant cells that tolerate off-target edits and have an enhanced risk for mutagenesis [240,241]. This insight further highlights that screening of engineered cells is imperative for therapeutic applications to confirm that Cas9-mediated cleavage has not compromised the p53 function or that the engineered cells do not carry immunogenicity risks, which is possible within the context of ex vivo gene editing. The limitation of ex vivo gene editing is its impracticality for certain disease conditions in which the target cells are distributed in tissues and organs that cannot be isolated and subsequently transplanted, such as the lungs, or are localized in multiple organ systems. In these cases, systemic delivery of gene-editing tools using in vivo delivery strategies is unavoidable. Therefore, more studies are needed to improve the safety of nonviral system delivery approaches for gene editing.

Considering the multitude of challenges, it is essential that the field of genome editing continues to make progress in developing novel approaches to deliver gene-editing reagents into target cells with high efficiency to further enhance the safety of gene editing and editing capabilities to unleash the full therapeutic potential of gene editing.

## Figures and Tables

**Table 1:** Delivery method comparison for gene editing.

Method	Delivery Material	Approach	Carrying Capacity	Advantages	Disadvantages
Adenoviruses	Double-stranded DNA	in vivo	7.5–30 kb	- Enables simultaneous packaging of CRISPR components - High transfection efficiency	- High immunogenicity
Adeno-associated viruses	single-stranded DNA	in vivo	4.8 kb	- Mild toxicity - Low immunogenicity - High transfection efficiency	- Limited packaging capacity - Immunogenicity risk still exists
Lentiviruses	single-stranded RNA	in vivo	8 kb	- High transduction efficiency - Enables simultaneous packaging of CRISPR components	- Potential insertional mutagenesis - Off-target effects due to persistent expression
Hydrodynamic delivery	DNA plasmid, ribonucleoprotein (RNP)	in vivo		- No need for viral vectors - Simple - Cost-effective	- Large volumes of gene solution required - Traumatic to tissues
Electroporation	DNA plasmid, mRNA, RNP	ex vivo		- High transfection efficiency - Viral free - Amenable to difficult to transduce cells - relatively fast - Delivery of transient forms of nucleases (i.e., Cas9 RNP)	- Low cell viability - high cost for reagents and cuvettes
Lipid nanoparticles	DNA plasmid, mRNA, RNP	in vivo		- Viral free - Low cost and no instrument required	- Toxicity concerns - Not efficient delivery for some cell types
Cell-penetrating peptides	Protein, RNP	in vivo		- Viral free - delivery of transient forms of Cas9	- Immunogenicity risks - inefficient delivery and low editing
